# Does dropout from school matter in taking antenatal care visits among women in Bangladesh? An application of marginalized poisson-poisson mixture model

**DOI:** 10.1186/s12884-022-04794-w

**Published:** 2022-06-13

**Authors:** M. Ershadul Haque, Taslim Sazzad Mallick, Wasimul Bari

**Affiliations:** Department of Statistics, University of Dhaka, Dhaka, 1000 Bangladesh

**Keywords:** Antenatal care, BDHS, Count model, Dropout, Marginalized, Mixture

## Abstract

**Background:**

There exists a lack of research in explaining the link between dropout from school and antenatal care (ANC) visits of women during pregnancy in Bangladesh. The aim of this study is to investigate how the drop out from school influences the ANC visits after controlling the relevant covariates using an appropriate count regression model.

**Methods:**

The association between the explanatory variables and the outcome of interest, ANC visits, have been performed using one-way analysis of variance/independent sample t-test. To examine the adjusted effects of covariates on the marginal mean of count data, Marginalized Poison-Poisson mixture regression model has been fitted.

**Results:**

The estimated incidence rate of antenatal care visits was 10.6% lower for the mothers who were not continued their education after marriage but had at least 10 years of schooling (*p*-value <0.01) and 20.2% lower for the drop-outed mothers (*p*-value <0.01) than the mothers who got continued their education after marriage.

**Conclusions:**

To ensure the WHO recommended 8+ ANC visits for the pregnant women of Bangladesh, it is essential to promote maternal education so that at least ten years of schooling should be completed by a woman and dropout from school after marriage should be prevented.

## Introduction

Antenatal care (ANC) is an important element of persistence care that a mother receives before and during pregnancy, at the time of childbirth, and the postnatal period. The aim of ANC is to detect any pregnancy complications, take immediate necessary steps for solving such complications, and prepare mother for safe and healthy birth. It has both direct and indirect influences on the survival and health of the mother as well as newborn [1, 2].

The World Health Organization (WHO) recently recommended for at least eight ANC visits for a woman during a pregnancy period [2]. The percentage of pregnant women receiving at least four ANC visits during a pregnancy period in Bangladesh has been increasing over the past two decades, from 6% in 1993–94 to 47% in 2017–18 [3]. Despite this substantial progress in ANC services, only about 11.5% of sampled women received WHO recommended 8+ ANC visits during November 2014-March 2018 in Bangladesh [3]. Therefore, to achieve the health related Sustainable Development Goals, it is essential to make access to quality ANC service easy for the pregnant mother and proper monitoring system is required to observe whether this service implemented throughout the country [4].

Education is one of the important determinants of good health as heath disparities can be determined by educational disparities [5, 6]. Mother’s education is considered as a crucial factor for preventing and treating poor health outcomes and effective use of health care services [7]. To ensure the best health practice, public policies should focus on getting the best attainable education for women among all socioeconomic classes [8] as there exist extreme gender differentiation in economic roles, lower parental investments in daughters than in sons, and significant restrictions on girls’ public mobility [9]. The women having more education are more likely to receive ANC services. Educated women understand the benefits of taking frequent ANC services as they are more knowledgeable about reproductive health care [10, 11]. School dropout should be considered as a public heath problem because education is a strong predictor of long-term health and dropouts have poorer health outcome [12, 13]. Although school dropout and child marriage are interrelated outcomes that have an enormous adverse impact on adolescent girls health and wellbeing as well as on their progeny, most of the parents of Bangladesh think that marriage and stop going school is the better option for their daughter’s prosperity [14]. The complex relationship between girl’s high school dropout and risky adolescent behavior suggests that schoolgirl dropout due to marriage has been associated with high risk of subsequent teen pregnancy [15–17] and teen pregnancy can lead to medical complications for the mother and infant [18].

There is a lack of research in explaining the link between whether or not a woman continued their education after marriage and ANC visits. Although the ANC services in Bangladesh has increasing trend over the decades, access to ANC services should be improved drastically to achieve the WHO recommended positive pregnancy outcome and the health Sustainable Development Goals. Therefore, the aim of the current study is to investigate the determinants of the frequency of ANC visits of women during their most recent pregnancy period in Bangladesh by applying an appropriate count regression model giving special emphasis on how the schoolgirl dropout influence the response of interest.

In practice, it may happen that sample count data observations may arise from two or more populations. Analyses of such data using standard or zero augmented count models may result in misleading conclusion [19, 20]. To overcome this difficulty, it may require to analyze the data taking mixture of populations into account [21]. Therefore, it is necessary to check whether count data come from mixture of populations. Moreover, the significance of inference regarding the marginal mean is well documented in many studies under mixture model setup [22–25]. Although finite mixtures of the standard count models have the advantage of describing over-dispersed count data, inferences regarding the overall exposure effects on marginal mean cannot be made from these model [22, 24].

The regression parameters for the marginal means can also be estimated sometimes indirectly by using the latent class parameters but such parameters cannot elucidate the link between the covariates and the population-wide parameters properly [26]. For this purpose, Benecha et al. [26] proposed marginally-specified mean models for mixtures of two count distributions to facilitate the maximum likelihood estimation where the mixing proportion have been included in the model along with the parameters for marginal mean. These are marginalized Poisson-Poisson (MPois-Pois) model and marginalized negative binomial-Poisson (MNB-Pois) model. In this study, an attempt has been made to draw inferences about the overall exposure effects on marginal means of ANC visits by using the MPois-Pois model.

## Data and methods

### Data

In this study, a nationwide data extracted from Bangladesh Demographic and Health Survey (BDHS) 2017–2018 have been utilized to analyze the number of ANC visits taken by a woman during her pregnancy period. The survey was based on a two-stage stratified sample of households. In the first stage of sampling, 250 enumeration areas (EAs) from urban and 425 from rural areas were selected. In the second stage, an average of 30 households per EA were selected. Based on the design, A total of 20127 ever married women of reproductive age were interviewed to collect data on fertility, family planning along with socioeconomic and demographic characteristics. BDHS data also provides information on several aspect of maternal and newborn health, including antenatal care (ANC). The information regarding number of ANC that a woman receives during pregnancy was collected from 5012 women who gave their most recent birth in the 3 years preceding the survey. Finally, a sample of 4941 women was included in the analysis as they provide complete information about all the variables considered in the study.

### Variables

The outcome variable is ‘the number of ANC visits’ that a woman received during pregnancy for her most recent birth. The exposure variable ‘study continuity status’ is categorized into three categories. The categories of this variable were created based on ‘the number of years of schooling of a woman’ and ‘whether she studying or attending school just before getting married’ or ‘whether she continued studying after marriage’. These categories are

- continued studying after marriage: continued studying after marriage and total number of schooling ≥10 years;
- not continued but having ≥10 years schooling: ‘stop attending school before getting married or stopped continuing study after marriage’ but having ≥10 years of schooling;
- drop-outed: having <10 years of schooling.

Therefore, the women who could not reach at 10 academic years of schooling is considered as ‘schoolgirl dropout’ in this study. Note that ‘dropout’ category consists of three mutually exclusive classes: ‘stop attending school before getting married’, ‘stopped continuing study after marriage’, ‘continued studying after marriage’. The effects of some other covariates were controlled in this study based on some available literatures [10, 27, 28]. These variables are: place of residence (urban, rural); exposed to any of the three media newspaper/magazine, radio, and television at least once a week (yes, no); mother’s age at birth in years (<20, 20-29, ≥30); difference between husband and wife age in years (non-positive, 1-5, 6-10, >10); number of decisions woman participated out of three major decisions regarding her own health care, large household purchases, and visits to family/relatives (none, 1-2, all 3); number of reasons a woman justified beating by her husband out of five regarding if she goes out without telling husband, neglects the children, argues with husband, refuses to have sex with husband, and burns the food (not at all, 1-2, 3-5); wealth index (poor, middle, rich) was created by using the ranked wealth index factor scores and then dividing the ranking into three equal categories, each comprising 33.33 percent of the sampled households; and order of birth of the index pregnancy (1, 2, 3, ≥4).

### Statistical analyses

In this study, all the explanatory variables considered are categorical and the outcome variable of interest is the number of ANC visits during a pregnancy, which is discrete in nature. Therefore, we have performed descriptive statistics by computing percent distribution for the explanatory variables and by mean and standard deviation for the outcome variable. As all the explanatory variables are categorical, in order to draw inference about the association between the explanatory variables and the count outcome of interest we have performed one-way analysis of variance (ANOVA)/independent sample t-test. To justify whether data arise from a single or two different populations, we have computed the mixing proportion of the count variable using the marginalized Poisson-Poisson (MPois-Pois) mixture model [26] in the absence of covariates. Finally, to examine the adjusted effects of covariates on the marginal mean of count data, MPois-Pois mixture model has been used along with computing incidence density ratio (IDR).

### Model

The source population from which the count data have been collected is assumed to be partitioned into two latent subpopulations having Poisson distributions with mean *μ*_1_ and *μ*_2_, respectively. Let *Y*_1_,...,*Y*_*n*_ be a random sample of size *n*. Following [21], the Poisson-Poisson mixture probability distribution of *Y*_*i*_(*i*=1,...,*n*) can be written as

1 $$ f(Y_{i}=y_{i}|\pi, \boldsymbol{\mu}_{i}^{\prime})=\pi\frac{e^{-\mu_{1i}}\mu_{1i}^{y_{i}}}{y_{i}!}+(1-\pi)\frac{e^{-\mu_{2i}}\mu_{2i}^{y_{i}}}{y_{i}!},  $$

where ***μ***_*i*_=(*μ*_*i*1_,*μ*_*i*2_)^′^ and *π* is the mixing proportion. Hence, the marginal mean and variance of *Y*_*i*_ can be given respectively as

$$\begin{aligned} E(Y_{i})&=\mu_{i}=\pi\mu_{1i}+(1-\pi)\mu_{2i}\quad\text{and} \\ \text{Var}(Y_{i})&=\sigma^{2}_{i}=\mu_{i}+\pi\left(1-\pi\right)\left(\mu_{1i}-\mu_{2i}\right)^{2}. \end{aligned} $$

One may obtain the MPois-Pois mixture model by replacing *μ*_2*i*_ with (1−*π*)^−1^(*μ*_*i*_−*πμ*_1*i*_) in (1) [26]. To introduce the covariates into the marginalized model, the following specifications have been considered

$$\log(\mu_{i})=\boldsymbol{x}_{i}^{\prime}\boldsymbol{\beta},\quad \log(\mu_{1i})=\boldsymbol{z}_{i}^{\prime}\boldsymbol{\alpha},\quad \text{logit}(\pi)=\tau, $$

where $ \boldsymbol {\mathcalligra {x}}_{\boldsymbol {\mathcalligra {i}}}$ and ***β*** respectively are *p*_1_×1 vector of covariates and regression parameters associated with the marginal mean *μ*_*i*_ and $ \boldsymbol {\mathcalligra {z}}_{\boldsymbol {\mathcalligra {i}}}$ and ***α*** respectively are *p*_2_×1 vector of covariates and regression parameters associated with the subpopulation mean *μ*_1*i*_ and −*∞*<*τ*<*∞* is a constant. It can be permissible to use same covariates for both classes (i.e., $\boldsymbol {\mathcalligra {x}}_{\boldsymbol {\mathcalligra {i}}}=\boldsymbol {\mathcalligra {z}}_{\boldsymbol {\mathcalligra {i}}}$) with *p*_1_=*p*_2_=*p*.

For the jth covariate in a MPois-Pois model where $\boldsymbol {\mathcalligra {x}}_{\boldsymbol {\mathcalligra {i}}}=\boldsymbol {\mathcalligra {z}}_{\boldsymbol {\mathcalligra {i}}}$, IDR, the ratio of means for a one-unit increase in *x*_*ij*_, is obtained as follows

$$\frac{E(Y_{i}|x_{ij}=k+1,\tilde{\boldsymbol{\mathcalligra{x}}}_{\boldsymbol{\mathcalligra{i}}})}{E(Y_{i}|x_{ij}=k,\tilde{\boldsymbol{\mathcalligra{x}}}_{\boldsymbol{\mathcalligra{i}}})}=\exp(\beta_{j}); $$

where $\tilde {\boldsymbol {\mathcalligra {x}}}_{\boldsymbol {\mathcalligra {i}}}$ indicates all covariates except *x*_*ij*_. The primary interest is to estimate the regression parameters (***β***) of the marginal mean (*μ*_*i*_) whereas the nuisance parameters ***α*** and *τ* need to be estimated to facilitate the maximum likelihood estimation of ***β***. The likelihood function of MPois-Pois model is given as follows

$${{}\begin{aligned} L(\boldsymbol{\tau}, \boldsymbol{\alpha}, \boldsymbol{\beta}|\boldsymbol{y})=\prod\limits_{i=1}^{n}&\frac{1}{(1+e^{\tau})y_{i}!} \left\{e^{\tau}\exp(-e^{\boldsymbol{z}_{\boldsymbol{i}}^{\prime}\boldsymbol{\alpha}}) e^{\boldsymbol{z}_{\boldsymbol{i}}^{\boldsymbol{\prime}}\boldsymbol{\alpha}y_{i}} + \right.\\ &\qquad \left.e^{-\eta(\tau,\boldsymbol{\beta},\boldsymbol{\alpha}\boldsymbol{;x}_{\boldsymbol{i}},\boldsymbol{z}_{\boldsymbol{i}})}\eta(\tau,\boldsymbol{\beta,\alpha;x_{i},z_{i}})^{y_{i}}\right\}, \end{aligned}} $$

where $ \eta (\tau,\boldsymbol {\beta },\boldsymbol {\alpha }\boldsymbol {;x}_{\boldsymbol {i}}, \boldsymbol {z}_{\boldsymbol {i}})=e^{\boldsymbol {x}_{\boldsymbol {i}}^{\boldsymbol {\prime }}\boldsymbol {\beta }}(1+e^{\tau })-e^{\tau } e^{\boldsymbol {z}_{\boldsymbol {i}}^{\boldsymbol {\prime }}\boldsymbol {\alpha }}$. With carefully chosen starting values, parameters of MPois-Pois can be estimated by the use of quasi-Newton optimization method. The quasi-Newton optimization can be implemented by SAS ‘nlmixed’ or R ‘optim’ function to get estimates from above likelihood function. Starting values for *τ* and ***α*** can be obtained by EM algorithm from Poisson-Poisson mixture model. Also, the initial values of ***β*** can be obtained by fitting a marginalized zero-inflated Poisson (MZIP) model [26].

## Results

To analyse the count variable of interest, the number of ANC visits during pregnancy, we have considered 4,941 women who gave the index birth in the three years preceding the survey after adjusting for missing values. The mean, standard deviation, minimum and maximum of the number of ANC visits were 3.93, 2.88, 0 and 20, respectively. It was found that the frequency of ANC visits in Bangladesh arises from mixture of two unobserved populations with proportions 0.55 and 0.45 in the absence of covariates. The mean number of ANC visits for each category of explanatory variables along with its standard errors, 95% confidence interval (CI) and the percent distribution of respondents in each category of selected covariates are presented in Table 1.

**Table 1:** The mean, standard error(SE) and 95% confidence interval (CI) for number of ANC visits during pregnancy of women in Bangladesh by some selected socioeconomic and demographic characteristics, BDHS 2017

Variables	n(%)	Mean	SE	95% CI
Study continuity status ^∗∗∗^				
Continued after marriage	552(11.2)	5.57	0.128	5.32-5.82
Not continued but had ≥10 years schooling	609(12.3)	4.89	0.115	4.67-5.12
Drop-outed	3780(76.5)	3.54	0.045	3.45-3.62
Place of residence ^∗∗∗^				
Urban	1696(34.3)	4.71	0.076	4.56-4.86
Rural	3245(65.7)	3.52	0.046	3.43-3.62
Exposed to media ^∗∗∗^				
No	2265(45.8)	3.07	0.052	2.97-3.17
Yes	2676(54.2)	4.66	0.058	4.55-4.77
Mother’s age at birth (years) ^∗∗∗^				
<20	1377(27.9)	3.79	0.074	3.64-3.93
20-29	2816(57.0)	4.05	0.055	3.94-4.16
≥30	748(15.1)	3.74	0.108	3.53-3.95
Difference between husband and wife age (years) ^∗∗∗^				
Non-positive	55(1.1)	5.22	0.514	4.21-6.23
1-5	1602(32.4)	3.88	0.071	3.74-4.02
6-10	2098(42.5)	3.92	0.065	3.79-4.04
>10	1186(24.0)	3.97	0.079	3.81-4.12
Number of decisions woman participated				
None	747(15.1)	3.76	0.103	3.55-3.96
1-2	1492(30.2)	3.92	0.071	3.78-4.06
All 3	2702(54.7)	3.99	0.057	3.87-4.10
Number of reasons wife beating justified ^∗∗∗^				
Not at all	4055(82.1)	4.07	0.046	3.98-4.17
1-2	708(14.3)	3.33	0.095	3.14-3.52
3-5	178(3.6)	3.03	0.188	2.66-3.40
Wealth index ^∗∗∗^				
Poor	1823(36.9)	3.00	0.060	2.88-3.11
Middle	1588(32.1)	3.92	0.069	3.79-4.06
Rich	1530(31.0)	5.05	0.077	4.90-5.20
Birth Order ^∗∗∗^				
1	1870(37.9)	4.37	0.066	4.24-4.50
2	1623(32.8)	4.03	0.073	3.89-4.18
3	848(17.2)	3.68	0.097	3.49-3.87
≥4	600(12.1)	2.64	0.100	2.45-2.84

***p-value <0.01; **p-value <0.05; *p-value <0.10

From Table 1 it was found that every three out of four mothers (76.5%) were drop-outed from school, 12.3% were completed at least 10 years of schooling but stopped education after marriage, whereas 11.2% had continued their education after marriage and reached at least 10 years of schooling. About two-third of the mothers (65.7%) were sampled from rural area and rest were from urban area. Of the mothers, about 54.2% were exposed to any of the three media at least once a week. Most of the mothers (57.0%) had given their index birth at age 20–29 years, 15.1% at age 30–39 years, and a little more than one-forth (27.9%) at age below 20 years. It was found that 24.0% couple had age gap (difference between husband and wife age) more than 10 years, 42.5% had 6–10 years, 32.4% had 1–5 years, whereas for 1.1% couple husband age did not exceed wife age. Most of the women (54.7%) participated in all three major decisions regarding themselves, 30.2% participated in 1–2 and 15.1% did not participate in any such decisions. Most of the women (82.1%) had justified none of all five reasons of wife beating, 14.3% had justified 1–2 reasons, whereas only 3.6% had justified 3–5 reasons. Women were distributed over the categories of wealth index almost equally. Most of the mothers (37.9%) had given their index birth as first birth, 32.8% as second birth, 17.2% as third birth and for rest of the mothers child birth order were 4 or more.

The exposure variable ‘study continuity status’ was found to have a significant association (*p*-value <0.01) with the ANC taking behavior. The average rate of ANC visits was largest (5.57) for the mothers if they had been continued their education after marriage and smallest (3.54) for those who drop-outed from school. The average number of ANC visits was significantly (*p*-value <0.01) higher (4.71) for the urban mothers than the rural (3.52). It was also higher (4.66) for the mothers who were exposed any of the three media at least once a week than who were not exposed (3.07) with *p*-value <0.01. The mean ANC visits was highest (4.05) for the mothers whose age at birth for the index child was 20–29 years whereas it was 3.79 and 3.74 for mother’s age at birth below 20 years and at least 30 years respectively, the result was statistically significant with *p*-value <0.01. The average ANC visits was significantly (*p*-value <0.01) higher (5.22) for the mothers whose partner age did not exceed their age, and this average seems to be similar for the other categories of ‘difference between husband and wife age’. The averages did not differ significantly among different categories of ‘number of decisions woman participated’. The average rate of visits was inversely related with the number of reasons a woman justified beating by her husband (*p*-value <0.01) and also with the birth order number (*p*-value <0.01). However, it was positively related with the wealth quintiles (*p*-value <0.01).

Since in the absence of covariates one of the proportions of mixture was found as 0.55, we have fitted the MPois-Pois model to analyse the number of ANC visits taken by women during a pregnancy period in Bangladesh. As our main interest is to estimate the marginal parameters, the $ \hat {\boldsymbol {\beta }} $ along with IDR are presented in Table 2. From Table 2 it was found that, the estimated incidence rate of ANC visits was 10.6% lower for the mothers who were not continued their education after marriage but had at least 10 years at schooling (*p*-value <0.01) and 20.2% lower who drop-outed from studying (*p*-value <0.01) than the mothers who got continued their education after marriage. The IDR of ANC visits was 1.12 for urban to rural mothers (*p*-value <0.01), 1.24 for exposed media at least once a week to unexposed mothers (*p*-value <0.01). The rate of ANC visits was 7.7% lower for mothers who gave their index birth below 20 years of age (*p*-value <0.01) and 5.3% higher for mothers who gave their index birth after or at 30 years of age (*p*-value <0.10) than those who gave birth during age 20–29 years. The IDR of ANC visits was 1.26 for the women who are not younger than their husbands to the women whose age lag behind 1–5 years than their husbands (*p*-value <0.05), this IDR was statistically insignificant for the other categories of ‘difference between husband and wife age’. The rates of ANC visits were statistically insignificant among all categories of ‘number of decisions woman participated’. The IDR of ANC visits was 0.88 for the mothers who justified beating by their partners for 1–2 reasons to the mothers who never justified for any of the five reasons (*p*-value <0.01), this ratio was about the same for the mothers who justified beating by their partners for 3–5 reasons (*p*-value <0.05). The rate of ANC visit was 13.8% higher for the mothers from middle-wealth households than the poor households, 24.1% higher for the mothers from rich households than the poor households, both the results were statistically significant with *p*-value <0.01. As the birth order of index child increases, the mothers were significantly less likely to take ANC care during their pregnancy. For example, the rate of ANC visits of mothers during their pregnancy were 7.2%, 10.9% and 30.9% less likely for the second birth, third birth and forth or upper order birth respectively compared to the first birth. It was also seen that the marginal mean parameters were estimated from mixture of two latent subpopulations with the proportions 0.61 and 0.39 after adjusting the covariates.

**Table 2:** The estimated marginal parameters ($ \hat {\beta } $), standard errors (SE), and *p*-values, 95% CI for regression parameters, and IDR under MPois-Pois mixture model on number of ANC visits during a pregnancy, BDHS 2017

	$ \hat {\beta } $	SE	z-value	*p*-value	95% CI	IDR
Intercept	1.359	0.042	32.708	<0.001	-	3.893
Study continuity status						
Continued after marriage (ref)						
Not continued but had ≥10 years schooling	-0.112	0.030	-3.755	<0.001	-0.171- -0.053	0.894
Drop-outed	-0.225	0.026	-8.679	<0.001	-0.276- -0.174	0.798
Place of residence						
Rural (ref)						
Urban	0.113	0.021	5.457	<0.001	0.072-0.154	1.120
Exposed to media						
No (ref)						
Yes	0.218	0.022	10.072	<0.001	0.175-0.261	1.243
Mother’s age at birth (years)						
<20	-0.080	0.025	-3.226	0.001	-0.129- -0.031	0.923
20-29 (ref)						
≥30	0.052	0.031	1.691	0.091	-0.009- 0.113	1.053
Difference between husband and wife age (years)						
Non-positive	0.228	0.098	2.328	0.020	0.036-0.420	1.256
1-5 (ref)						
6-10	-0.002	0.021	-0.110	0.913	-0.043- 0.039	0.998
>10	0.011	0.025	0.432	0.666	-0.038- 0.060	1.011
Number of decisions woman participated						
None (ref)						
1-2	0.002	0.029	0.079	0.937	-0.055- 0.059	1.002
All 3	0.021	0.027	0.781	0.435	-0.032- 0.074	1.021
Number of reasons wife beating justified						
Not at all (ref)						
1-2	-0.126	0.028	-4.535	<0.001	-0.181- -0.071	0.881
3-5	-0.128	0.057	-2.221	0.026	-0.240- -0.016	0.880
Wealth index						
Poor (ref)						
Middle	0.129	0.025	5.082	<0.001	0.080-0.178	1.138
Rich	0.216	0.029	7.338	<0.001	0.159-0.273	1.241
Birth Order						
1 (ref)						
2	-0.075	0.024	-3.140	0.002	-0.122- -0.028	0.928
3	-0.116	0.032	-3.601	<0.001	-0.179- -0.053	0.891
≥4	-0.369	0.043	-8.492	<0.001	-0.453- -0.285	0.691
Mixing Proportion						
$\hat {\pi }$	0.610	0.049	12.472	<0.001	-	-

To asses the goodness of fit of the MPois-Pois, with selected covariates, the Poisson, negative binomial regression model have also fitted. It was found that the AIC values of Poisson, negative binomial and MPois-Pois were 23468, 22543, and 22470 respectively. It implies that the MPois-Pois is the best choice for fitting this data set.

## Discussion and conclusion

To analyze the count data to draw valid inference, it is require to check whether the data come from a population or from a mixture of populations. If the target population consist of mixture of populations, the estimates of latent class parameters of the regression model can be obtained by fitting a mixture model. However, it is sometimes difficult to get estimate of the regression parameters for marginal means from the mixture model setup. Also, the interpretation in terms of IDR for the population-wide parameters can not be possible from such model. The marginalized mixture model can be utilized under these circumstance for drawing inferences about the whole population. The objective of this study is to explore the effect of schoolgirl dropout due to marriage on the number of ANC visits in Bangladesh (inference regarding the population-wide parameters). From the results, we have observed that the population count data can be regarded a mixture of two latent count distributions with proportions 0.55 and 0.45 in the absence of covariates. Therefore, a marginalized mixture model, the MPois-Pois model, has been utilized to meet the study objective.

Although tremendous successes has been documented for ANC service of the pregnant women, Bangladesh is still far away to ensure the WHO recommended 8+ ANC visits for the pregnant women. Receiving heath care service during pregnancy is associated with organization, accessibility, standard, value, health beliefs as well as some socio-demographic factors [27]. Maternal health care programme in Bangladesh has been confronted many challenges including, accessibility, lack of equity, lack of public health facilities, scarcity of skilled workforce and inadequate financial resource allocation [29]. To identify the obstacles for receiving ANC services, it is necessary to undertake some comprehensive research studies. This study aimed to identify some socio-demographic factors with a special emphasis to the schoolgirl dropout due to marriage that are associated with the frequency of ANC visits in Bangladesh.

Researchers were investigated which of the socioeconomic and demographic factors are responsible for the frequency of ANC visits women took during their pregnancy period in Bangladesh in some of the previous studies [10, 27, 28, 30]. Although they were included education as an independent variable and found that it was an important determinants of frequency of ANC visits, we have considered ‘study continuity status after marriage’ as the exposure variable in this study. The results revealed that schoolgirl dropout have tremendous negative influence on the rate of receiving ANC services of the pregnant women in Bangladesh. Consistent with findings of the aforementioned previous studies, this study found that women who are living in urban area, possessing higher wealth index and exposed to media have higher rate of receiving ANC visits.

Hossain et al. [28] found that the frequency of ANC visits was less for women aged 20 years or below compare to women aged 20–35 years at the time of delivery. Besides, Kabir and Islam [30] has found the odds of receiving ≥4 ANC services was low if mother’s age at conception is ≥35 years. However, we had found that the rate of receiving ANC services was smaller for women aged below 20 years but higher for women aged 30 years or more than whose age were 20–29 years at the time of delivery. In this study, we have found that higher order of birth was associated with lower rate of ANC visits. This findings are similar with previous studies [28, 30].

Bhowmik et al. [10] found that women who were more involved in making decision regarding own health care can significantly improve the frequency of ANC visits. However, Kabir and Islam [30] has found increased odds of taking ≥4 ANC visits if women and their husband jointly took decision regarding women own health care. But Hossain et al. [28] did not find any association between women empowerment and frequency of receiving ANC services. In this study we have considered three aspects of women empowerment as independent variables, These are: number of decisions woman participated out of three major decisions regarding themselves, number of reasons a woman justified beating by her husband out of five reasons of wife beating, and difference between husband and wife age (in years). Although the number of decisions woman participated was not associated with the frequency of ANC visits, increased number of reasons for beating justification was associated with lower rate of receiving ANC services. Also, the rate of receiving ANC services was higher for women who are not younger than their husbands.

Since adequate level of ANC visits during pregnancy period of women contributes to maintain proper maternal health and to protect the adverse pregnancy outcomes to a great extent, it is essential to make sure that every pregnant mother receives WHO recommended at least eight ANC visits. This can be accomplished by encouraging women to take sufficient number of ANC visits during their pregnancy period. This might be achieved in Bangladesh by promoting maternal education so that at least ten years of schooling should be completed by a woman and dropout from school after marriage should be prevented. Besides schoolgirl dropout, this study helps policy makers to identify other socio-demographic factors such as place of residence, media exposure, mother’s age at birth, women empowerment, wealth index and birth order that might uplift women for receiving more ANC visits during their pregnancy period.

## Limitations

The study has limitations as follows: (1) We used cross-sectional data to study the association between schoolgirl dropout and frequency of ANC visits during their pregnancy period by controlling some socio-demographic variables and therefore it would not be possible to asses the causal relationship between them; (2) Although the data have been collected using stratified cluster sampling technique, cluster-level variation has not been considered into the analysis. Further researchers can be carried out by taking this limitations into accout.

## Data Availability

The DHS dataset that was used in this study is freely available at https://www.dhsprogram.com/data/available-datasets.cfm once permission is sought and granted by DHS program authority.

## Abbreviations

ANC: Antenatal care
ANOVA: Analysis of variance
BDHS: Bangladesh Demographic and Health Survey
CI: Confidence interval
EA: Enumeration Area
IDR: Incidence density ratio
MNB-Pois: Marginalized negative binomial-Poisson
MPois-Pois: Marginalized Poisson-Poisson
MZIP: Marginalized zero-inflated Poisson
WHO: World Health Organization

## References

[1] World Health Organization and others. Annual report 2008. Technical report, Geneva: World Health Organization. 2009.

[2] World Health Organization and others. WHO recommendations on antenatal care for a positive pregnancy experience: executive summary. Technical report, World Health Organization. 2016. 28079998

[3] National Institute of Population Research and Training (NIPORT), and ICF. Bangladesh demographic and health survey 2017-18. 2020. Dhaka, Bangladesh, and Rockville, Maryland, USA: NIPORT and ICF.

[4] Jo Y, Alland K, Ali H, Mehra S, LeFevre AE, Pak SE, Shaikh S, Christian P, Labrique AB (2019). Antenatal care in rural bangladesh: current state of costs, content and recommendations for effective service delivery. BMC Health Serv Res.

[5] American Public Health Association and others. The dropout crisis: A public health problem and the role of school-based health care. 2011. https://www.shankerinstitute.org/sites/default/files/APHA4_article_DropOut_0914_FINAL3%20June%2012%202015.pdf.

[6] Freudenberg N, Ruglis J (2007). Peer reviewed: Reframing school dropout as a public health issue. Prev Chronic Dis.

[7] Smith LC, Ramakrishnan U, Ndiaye A, Haddad L, Martorell R (2003). The importance of women’s status for child nutrition in developing countries: international food policy research institute (ifpri) research report abstract 131. Food Nutr Bull.

[8] De Ridder KA, Pape K, Johnsen R, Holmen TL, Westin S, Bjørngaard JH (2013). Adolescent health and high school dropout: a prospective cohort study of 9000 norwegian adolescents (the young-hunt). PloS ONE.

[9] Mahmud S, Amin S (2006). Girls’ schooling and marriage in rural bangladesh. Children’s Lives and Schooling Across Societies.

[10] Bhowmik KR, Das S, Islam MA (2020). Modelling the number of antenatal care visits in bangladesh to determine the risk factors for reduced antenatal care attendance. PloS ONE.

[11] Guliani H, Sepehri A, Serieux J (2014). Determinants of prenatal care use: evidence from 32 low-income countries across asia, sub-saharan africa and latin america. Health Policy Plan.

[12] Lansford JE, Dodge KA, Pettit GS, Bates JE (2016). A public health perspective on school dropout and adult outcomes: A prospective study of risk and protective factors from age 5 to 27 years. J Adolesc Health.

[13] Pleis J, Ward B, Lucas J. Summary health statistics for us adults: National health interview survey, 2009. data from the national health interview survey. vital and health statistics. series 10, volume number 249. dhhs publication no.(phs) 2011-1577. Centers Dis Control Prev. 2010;:1–207. 21905346

[14] Arends-Kuenning M, Amin S (2001). Women’s capabilities and the right to education in bangladesh. Int J Polit Cult Soc.

[15] Marcotte DE (2013). High school dropout and teen childbearing. Econ Educ Rev.

[16] Rosenberg M, Pettifor A, Miller WC, Thirumurthy H, Emch M, Afolabi SA, Kahn K, Collinson M, Tollman S (2015). Relationship between school dropout and teen pregnancy among rural south african young women. Int J Epidemiol.

[17] Manlove J (1998). The influence of high school dropout and school disengagement on the risk of school-age pregnancy. J Res Adolesc.

[18] Gibbs CM, Wendt A, Peters S, Hogue CJ (2012). The impact of early age at first childbirth on maternal and infant health. Paediatr Perinat Epidemiol.

[19] Frühwirth-Schnatter S, Frèuhwirth-Schnatter S (2006). Finite Mixture and Markov Switching Models, vol 425.

[20] Wedel M, DeSarbo WS (1995). A mixture likelihood approach for generalized linear models. J Classif.

[21] Wang P, Puterman ML, Cockburn I, Le N (1996). Mixed poisson regression models with covariate dependent rates. Biometrics.

[22] Albert JM, Wang W, Nelson S (2014). Estimating overall exposure effects for zero-inflated regression models with application to dental caries. Stat Methods Med Res.

[23] Böhning D, Dietz E, Schlattmann P, Mendonca L, Kirchner U. J R Stat Soc Ser A (Stat Soc). 1999; 162(2):195–209.

[24] Preisser JS, Stamm JW, Long DL, Kincade ME (2012). Review and recommendations for zero-inflated count regression modeling of dental caries indices in epidemiological studies. Caries Res.

[25] Long DL, Preisser JS, Herring AH, Golin CE (2014). A marginalized zero-inflated poisson regression model with overall exposure effects. Stat Med.

[26] Benecha HK, Neelon B, Divaris K, Preisser JS (2017). Marginalized mixture models for count data from multiple source populations. J Stat Distrib Appl.

[27] Islam MM, Masud MS (2018). Determinants of frequency and contents of antenatal care visits in bangladesh: Assessing the extent of compliance with the who recommendations. PloS ONE.

[28] Hossain Z, Akter R, Sultana N, Kabir E (2020). Modelling zero-truncated overdispersed antenatal health care count data of women in bangladesh. PloS ONE.

[29] Islam A, Biswas T (2014). Health system in bangladesh: Challenges and opportunities. Am J Health Res.

[30] Kabir MR, Islam H (2019). Optimum utilization of antenatal care (anc 4) service is still a long way down the road in bangladesh: analysis of bangladesh demographic health survey data. Food Publ Health.

